# The role of cardiac magnetic resonance to address the treatment of choice for pulmonary valve replacement late after repair of Tetralogy of Fallot

**DOI:** 10.1186/1532-429X-16-S1-P132

**Published:** 2014-01-16

**Authors:** Benedetta Leonardi, Aurelio Secinaro, Sonia B Albanese, Nicoletta Cantarutti, Mara Pilati, Enrico Cetrano, Giacomo Pongiglione

**Affiliations:** 1 Department of Cardiology and Cardiac Surgery, Bambino Gesu' Children's Hospital IRRCCS, Rome, Rome, Italy

## Background

Severe right ventricular (RV) dilation and dysfunction due to chronic pulmonary regurgitation (PR) requires pulmonary valve replacement (PVR) late after repair of Tetralogy of Fallot (rTOF). Cardiac magnetic resonance (CMR) is the gold standard method to evaluate the pathophysiology after rTOF and the main tool to support the decision for PVR in asymptomatic patients. Given the various options available for PVR, we sought to evaluate the usefulness of CMR to address patients towards either a surgical or an interventional procedure.

## Methods

From March 2008 to August 2013, 84 patients (66 males, 18 females) underwent CMR study after either transanular or infundibular TOF repair. In addition to RV ventricle and function evaluation for PVR timing, a 3D navigator SSFP sequence was performed to assess pulmonary trunk (PT) length and dimensions at three levels (PV remnant, mid-portion, bifurcation) and coronary anatomy. The 3D navigator SSFP sequence was set in mid-diastole in 20 patients and in end-systole in the latter 64. Furthermore, PT expansion was assessed also by balanced SSFP cine views of the RVOT. Suitability for percutaneous treatment included maximum PT ranging from 19 to 27 mm and PT length >20 mm. Indications for perventricular injectable valve implantation consisted of PT diameter ranging from 15 to 31 mm and PT length >20 mm. Patients who did not meet such criteria were referred for surgery, as well as those with RVOT aneurisms, shape irregularity and/or significant systolic PT expansion.

## Results

Twenty patients were addressed to transcatheter PVR, which was unsuccessful in 10 cases due to the stent coronary compression on RVOT balloon occlusion test (10%) and/or excessive PT expansion (90%). The unsuccessful transcatheter PVR was due to the discrepancy of PT dimensions between 3D SSFP sequence set in diastole and catheterization. On the contrary, all cases addressed to PVR percutaneous on the basis of 3D SSFP sequence set in end-systole were successful. All remaining patients (88%) underwent surgery by means of bioprosthesis (54), homograft (12) and perventricular injectable valve (8) implantation. No deaths or complications were reported.

## Conclusions

3D navigator SSFP sequence set at end-systolic phase is more reliable in evaluating RV outflow tract measures and provides the best information to address patients to percutaneous.

## Funding

No disclosure to make.

**Figure 1:**
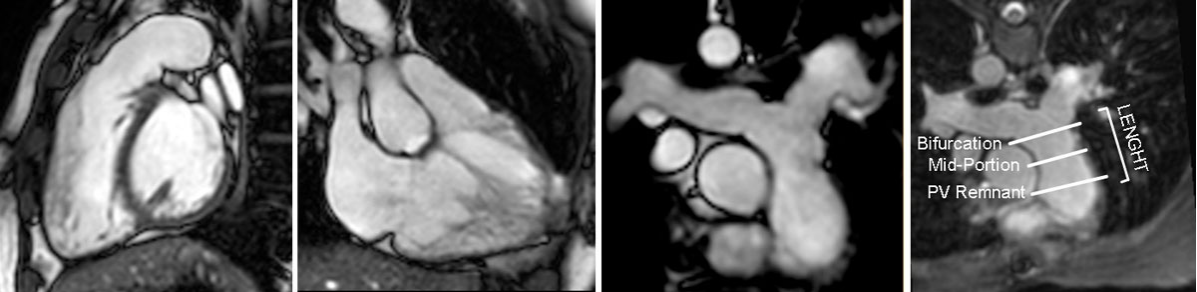
**The assessment of PT length and dimensions using, respectively, balanced SSFP cine and 3D SSFP navigator sequence**.

**Figure 2:**
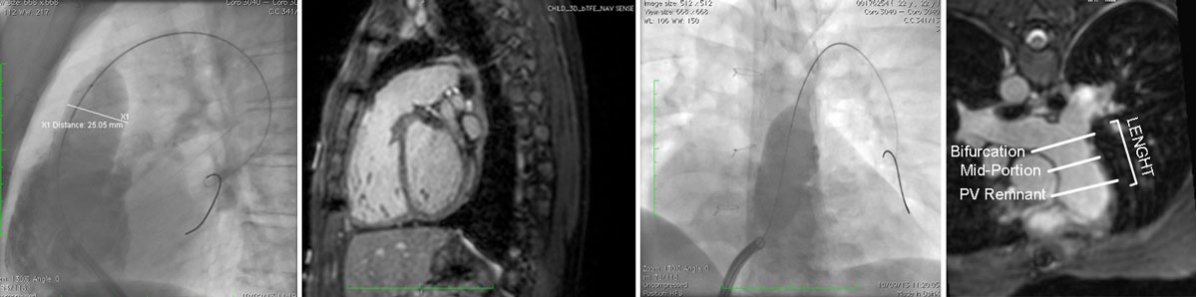
**RVOT in sagittal and coronal plane on catheterization and 3D navigator SSFP sequence**.

